# Cubic or Not Cubic? Combined Experimental and Computational Investigation of the Short-Range Order of Tin Halide Perovskites

**DOI:** 10.1021/acs.jpclett.3c00105

**Published:** 2023-02-21

**Authors:** Marta Morana, Julia Wiktor, Mauro Coduri, Rossella Chiara, Carlotta Giacobbe, Eleanor Lawrence Bright, Francesco Ambrosio, Filippo De Angelis, Lorenzo Malavasi

**Affiliations:** † Department of Earth Sciences, University of Firenze, Via G. La Pira 4, 50121 Firenze, Italy; ‡ Department of Physics, Chalmers University of Technology, 412 96 Goteborg, Sweden; ∥ Department of Chemistry and INSTM, Viale Taramelli 16, 27100 Pavia, Italy; ⊥ ESRF, 71 Avenue des Martyrs, 38000 Grenoble, France; # Department of Chemistry and Biology “A. Zambelli”, University of Salerno, Via Giovanni Paolo II 132, 84084 Fisciano, Salerno, Italy; @ Dipartimento di Scienze, University of Basilicata, Viale dell’Ateno Lucano, 10, 85100 Potenza, Italy; § Computational Laboratory for Hybrid/Organic Photovoltaics (CLHYO), Istituto CNR di Scienze e Tecnologie Chimiche “Giulio Natta” (CNR-SCITEC), Via Elce di Sotto 8, 06123 Perugia, Italy; ∇ Department of Chemistry, Biology and Biotechnology, University of Perugia, Via Elce di Sotto 8, 06123 Perugia, Italy; ● Department of Natural Sciences & Mathematics, College of Sciences & Human Studies, Prince Mohammad Bin Fahd University, Dhahran 34754, Saudi Arabia

## Abstract

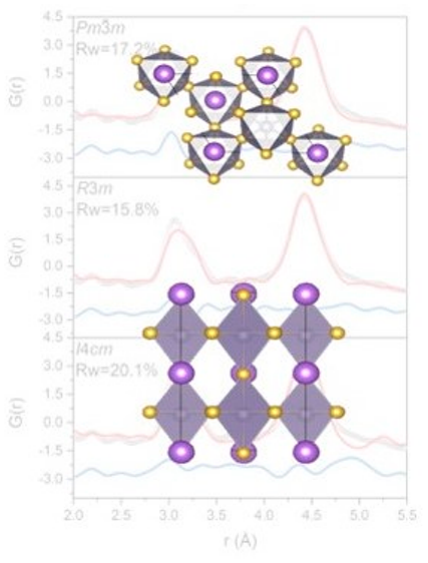

Tin-based metal halide perovskites with a composition of ASnX_3_ (where A= MA or FA and X = I or Br) have been investigated by means of X-ray total scattering techniques coupled to pair distribution function (PDF) analysis. These studies revealed that that none of the four perovskites has a cubic symmetry at the local scale and that a degree of increasing distortion is always present, in particular when the cation size is increased, i.e., from MA to FA, and the hardness of the anion is increased, i.e., from Br^–^ to I^–^. Electronic structure calculations provided good agreement with experimental band gaps for the four perovskites when local dynamical distortions were included in the calculations. The averaged structure obtained from molecular dynamics simulations was consistent with experimental local structures determined via X-ray PDF, thus highlighting the robustness of computational modeling and strengthening the correlation between experimental and computational results.

Metal halide perovskites (MHPs) are appealing materials because of their manifold applications, from photovoltaics to photocatalysis, and have attracted considerable attention in the past several years.^1−9^ These compounds have a general formula of ABX_3_, where A is a small organic or inorganic cation, such as methylammonium (CH_3_NH_3_^+^, MA), formamidinium [HC(NH_2_)_2_^+^, FA], or cesium (Cs), B is usually Pb, Sn, or Ge, and X is a halide, notably I, Br, or Cl. In recent years, together with the investigation of their functional properties, significant efforts have been directed toward a thorough comprehension of their crystal structure, the nature and type of possible phase transitions, and their phase stability under extreme conditions.^10−24^ Such a rich set of experimental and computational data led to a good understanding of the average structure of MHPs and provided the basis for structure–property correlations. However, the presence of weak chemical interactions when organic cations are present, namely, MA and FA, made the definition of the crystal structure by diffraction quite elusive, due to the presence of an order–disorder transition as a function of temperature.^25−27^ At the same time, the growing consensus about the presence of local octahedral distortions in MHPs, which may have an impact on the charge-carrier dynamics, makes a proper short-range order correlation between structure and properties key to fully addressing their microscopic description.^11,28−32^ Another interesting aspect influencing the local structure of MHPs is the possible activity of the lone pair, because group 14 elements have an s^2^p^0^ electronic configuration that can be potentially involved in symmetry-lowering distortions associated with the pseudo- or second-order Jahn–Teller effects.^28,33,34^ For example, it has been shown that the broad energy distribution of the photoluminescence maximum in polycrystalline samples of FASnI_3_ might be due to the activity of the lone pair of the Sn cation, which causes an off-centering of the cation, lowering the symmetry at the local level.^35−37^ Most of the efforts to date to understand the local structure of MHPs have been devoted to MAPbI_3_, for which the role of MA cation disorder was investigated in great detail both at the average and at the local structure level.^27,38^ Similar studies have also been carried out on other lead-based MHPs containing the MA or FA cation, namely, MAPbBr_3_, MAPbCl_3_, FAPbI_3_, and FAPbBr_3_.^36,39−41^ Because of these sets of local structure data, obtained through the use of total scattering methods coupled to pair distribution function (PDF) analysis, it was possible, for example, to unveil a common pattern of orthorhombic distortion at the local scale (1–10 Å) for the MAPbX_3_ compositions.^38−41^

On the contrary, the local structure landscape of lead-free MHPs is still poorly characterized, being limited to MASnI_3_ and FASnI_3_, despite the huge interest in replacing highly toxic lead with other elements like tin.^36^ For these two compositions, Laurita and co-workers investigated the short-range order by X-ray PDF analysis from room temperature (RT) to ∼480 K, highlighting dynamic, temperature-activated B-site cation off-centering displacements at and above RT as a consequence of the lone pair stereochemical activity.^36^ In addition, an enhancement of these displacements has been observed upon replacement of FA for MA or Br for I (which has been however determined in ref (36) on only lead-based perovskites). Such a trend has been correlated to the inherent instability of high-symmetry coordination for ns^2^p^0^ cations.^36^ To further confirm and better elucidate this preliminary evidence, it would be desirable to cover a more extended compositional range in terms of organic cations and halides and to broaden the temperature range by including low-temperature data to analyze a detailed *T* evolution of local and average structures.

In this study, we performed a systematic characterization of tin-based MHPs with a composition of ASnX_3_, where A = MA or FA and X = I or Br, from 80 to 360 K, by means of X-ray total scattering techniques coupled to PDF analysis. Data were collected on powdered samples sealed in capillaries at the Material Science Beamline ID 11 at the European Synchrotron Radiation Facility (ESRF). Details of the synthesis, data collection, and analysis can be found in the Supporting Information. Total scattering measurements made use of Bragg and diffuse scattering on an equal basis and were performed as in a regular powder diffraction measurement, and the PDF was obtained from the Fourier transform total scattering structure function, *S*(*Q*). Some information could be directly extracted from the PDF in a model-independent way because of its definition as the atom pair correlation function. From the peak position, it was possible to extract information about the bond length, the peak integrated intensities provided information about the coordination number, and, finally, the measurement of the peak width may allow extraction of information about thermal or static disorder. However, modeling of data, as in Rietveld refinement for diffraction data, reveals much more information than straight model-independent analysis.^42^

Before the short-range order of the ASnX_3_ perovskites was investigated, their average structure was studied by room-temperature Rietveld refinements of the diffraction patterns. The results are in good agreement with previous literature reports, with all four perovskites being cubic, in space group *Pm*3̅*m*, at room temperature and above.^43−45^ At the lowest investigated temperature, 80 K, all of the compounds adopt an orthorhombic symmetry except for MASnI_3_, which is tetragonal.^43−45^

Figure 1 reports the PDF data collected upon heating from 300 to 360 K. It is possible to observe a small peak broadening and a slight asymmetry that increase as *T* increases for all of the compositions, in particular for the first peak around 3 Å that is related to the Sn–X distances. The PDF features and this trend suggest a deviation from the ideal octahedral geometry occurring in a cubic perovskite in which only a single, symmetric peak is expected in that distance range.

**Figure 1:**
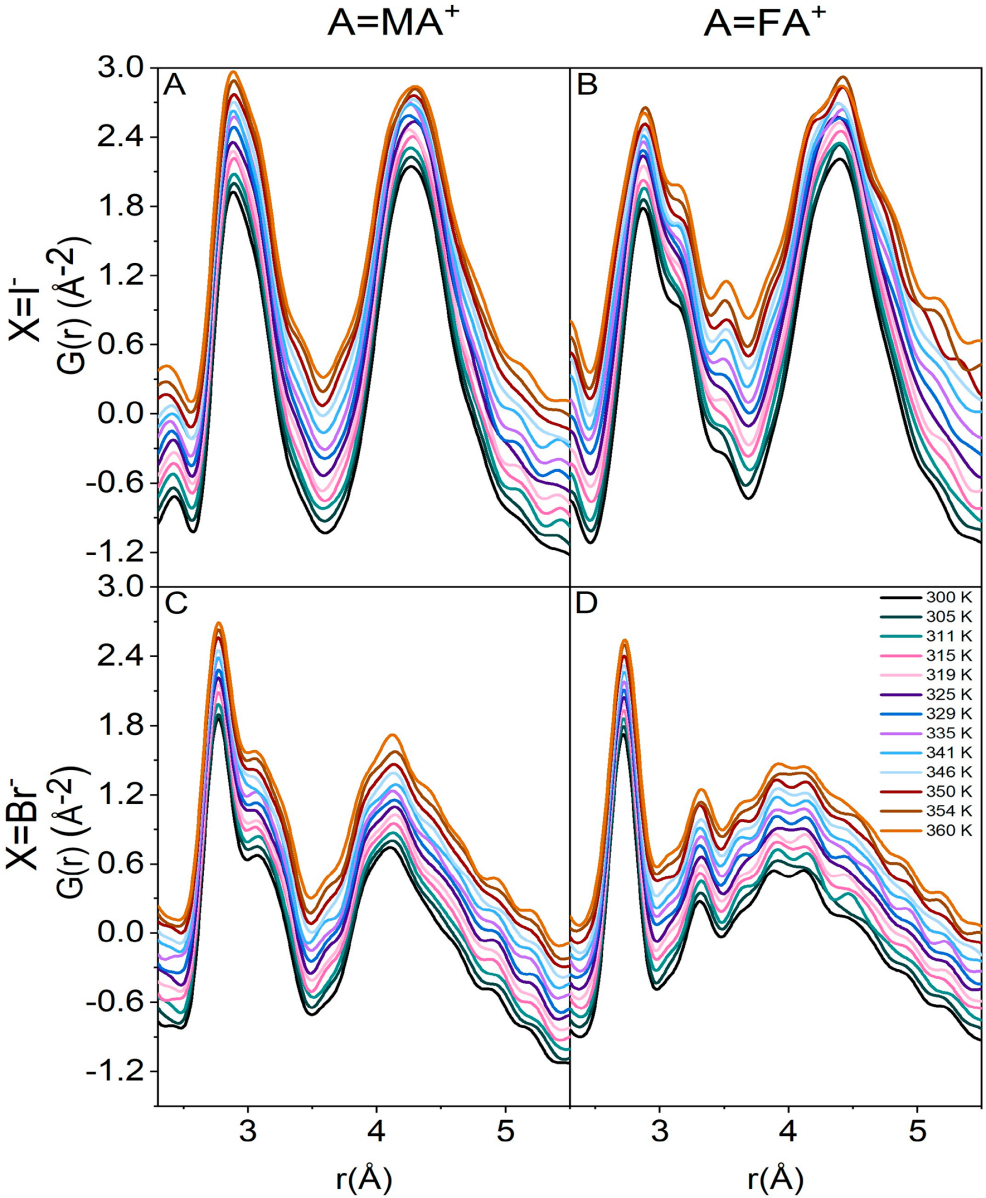
Overlay of the raw X-ray PDF data collected from 300 to 360 K from ASnX_3_ where A = MA or FA and X = I or Br: (A) MASnI_3_, (B) FASnI_3_, (C) MASnBr_3_, and (D) FASnBr_3_.

This effect increases with (i) the size of the A cation, with the influence of FA being stronger than that of MA, and (ii) the hardness of the anion (defined on the basis of the electronegativity and size according to ref (36)) that strengthens the interaction between the B-site s orbitals and the anion p states, and consequently the tendency for the activity of the lone pair, which results in a larger distortion of the octahedral environment.^36,46^ A similar trend was reported for MAPbI_3_, FAPbI_3_, MASnI_3_, FASnI_3_, MAPbBr_3_, and FAPbBr_3_.^36^ It is worth noting that peak broadening and asymmetry are most pronounced for FASnBr_3_ (Figure 1D), where the Sn–Br distances are spread over a large range up to the typical values of the Br–Br interactions. In fact, the large peak centered at 4 Å results from the contribution of the Sn–Br and Br–Br distances. The observed effects agree with the hypothesis that these compounds are subject to a dynamic off-centering of the B cation, which is thermally activated and thus enhanced by temperature.^36^ However, the role of the nature of the halide in the distortion induced by cation off-centering on a Sn-based perovskite has not yet been reported, the previous studies having been limited to iodide-containing materials or lead-based bromide perovskites.^36^

To understand the effect of the underlying distortion on the local structure of these materials, different models were tested against the RT X-ray PDF data. First, the aristotype perovskite structure in the *Pm*3̅*m* space group, which does not allow for octahedral tilting or displacement of the B cation, was taken into account. The RT PDF data fitted against this model led to a poor agreement for all of the samples (cf. Figure 2 and Figures S1 and S2; atom pairs involved in the PDF peaks in the figure are described in Figure 5). This is not completely surprising because none of the MHPs investigated so far adopts a cubic structure at the local level, but rather a lower-symmetry arrangement.^36,38−41^ Other tested models were the rhombohedral *R*3*m*, tetragonal *I*4*cm* and *I*4/*mcm*, and orthorhombic *Pnma* and *Pmc*2_1_ models. In the rhombohedral symmetry, there is no octahedral tilting, but the B cation is displaced along the [111] direction. The tetragonal models both introduce octahedral titling, but only *I*4*cm* allows for the displacement of the B-site cation along the [001] direction. The orthorhombic models induce a further distortion in the octahedra with the displacement of the B cation along a diad axis. In the case of MASnI_3_ and FASnI_3_ (Figure 2A,B), the *I*4*cm* and *R*3*m* models provide good fits of the RT data, and inspection of the different curves suggests that the rhombohedral distortion could better describe these compounds [*R*_w_ in all of the figures reporting fits of PDF data refers to the weighted *R* values calculated as the (weighted) square root of the difference between the observed and calculated *G*(*r*)].^47^

**Figure 2:**
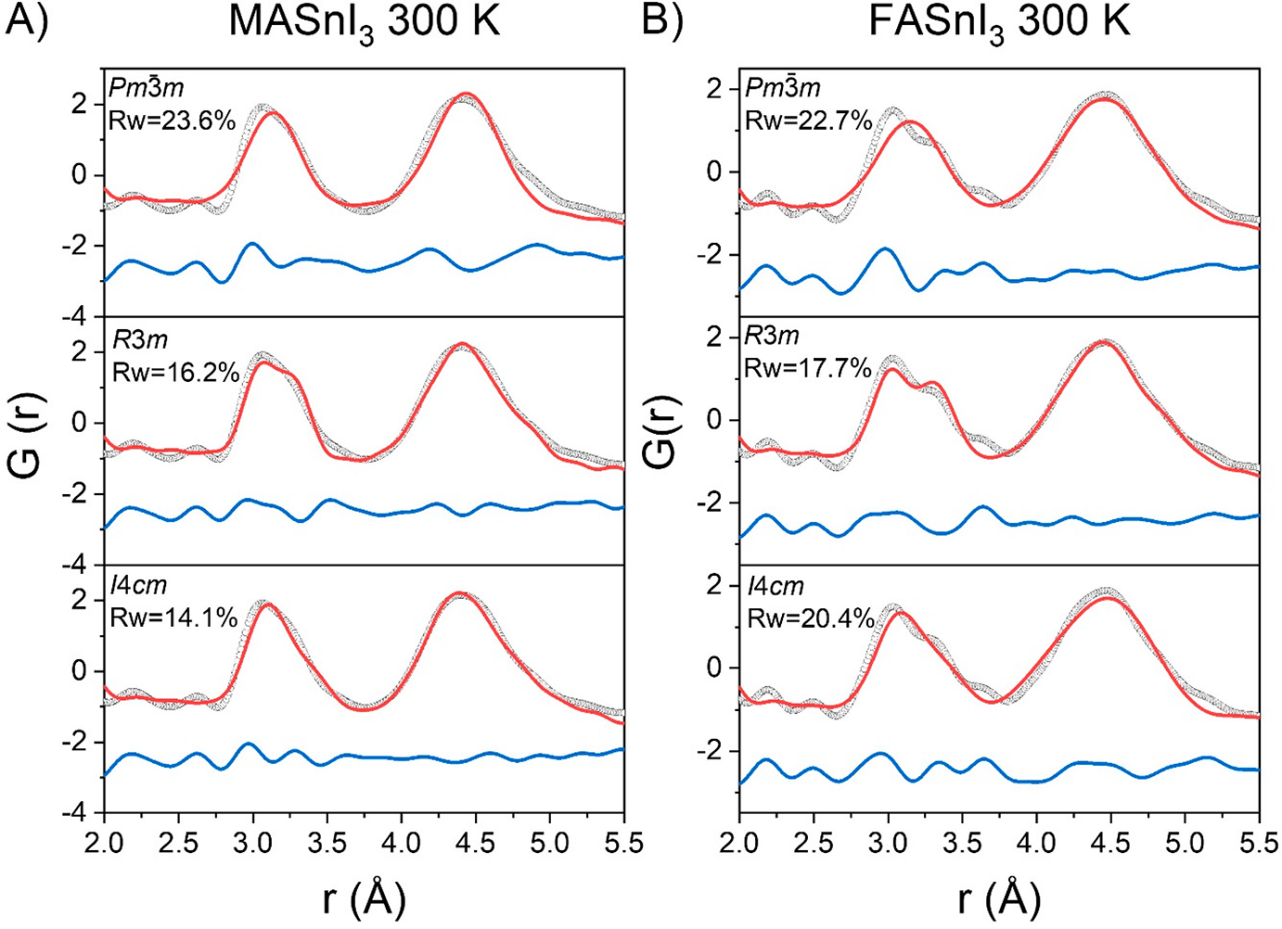
Fits of the X-ray PDF data at 300 K from 2.0 to 5.0 Å against the various space groups for (A) MASnI_3_ and (B) FASnI_3_: *Pm*3̅*m*, *R*3*m*, and *I*4*cm*. Gray dotted line, observed; red line, calculated; blue line, difference (shifted by −2.5 for ease of visualization).

The preference for the rhombohedral model is more pronounced for FASnI_3_ than for MASnI_3_, which is well reproduced also by the *I*4*cm* model (cf. Figure 2A), as in the case of MAPbI_3_.^38^ The rhombohedral model is fairly common in the world of perovskites such as in several well-known titanates (for the local and average structures), in CsSnBr_3_, and was also proposed to describe the local structure of FAPbBr_3_.^36,37,48−50^ The tetragonal and rhombohedral models induce a displacement of the B-site cation, suggesting that the off-centering of this cation is needed to properly describe the local structure and clearly indicating that the cubic model is not sufficient. The space groups of the respective low-temperature phases, *I*4/*mcm* for MASnI_3_ and *Pnma* for FASnI_3_, were also tested (Figure S3), because both MAPbBr_3_ and MAPbCl_3_ were reported to show the structure of their low-temperature phase in the local range at RT.^39−41^ However, none of these models led to an improvement in the fit to the experimental data. As already mentioned, the distortion increases moving from the iodide to the bromide compounds, making the choice of a good structural model more difficult for this second group. In the case of MASnBr_3_, the *Pm*3̅*m* model cannot reproduce the experimental trends, whereas the *R*3*m* and *I*4*cm* models give better results (Figure S1). However, the *Pmc*2_1_ model, the same structure of the low-temperature phase, provides the best fit of the clear splitting of the Sn–Br correlation (Figure 3A).

**Figure 3:**
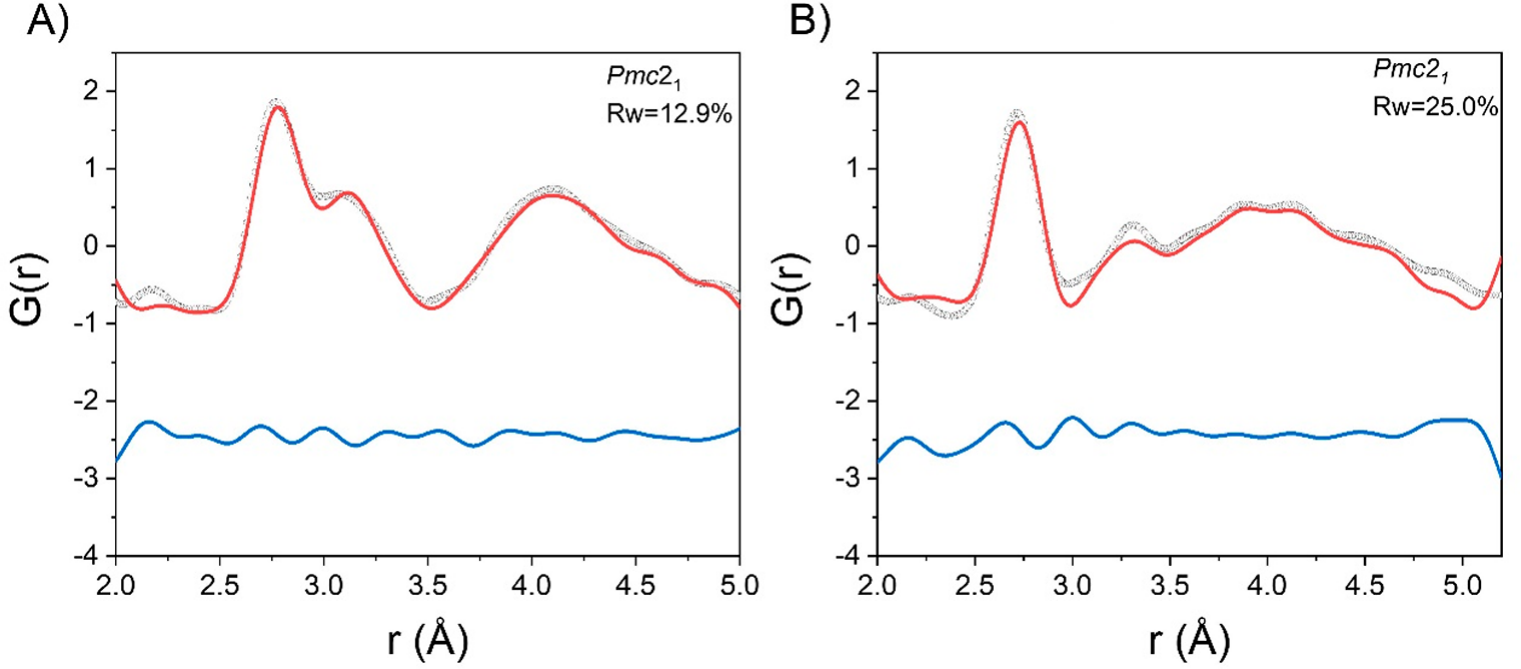
Fit of the X-ray PDF data at 300 K from 2.0 to 5.0 Å for (A) MASnBr_3_ and (B) FASnBr_3_ against the orthorhombic models. Gray dotted line, observed; red line, calculated; blue line, difference (shifted by −2.5 for ease of visualization).

All three models shown in Figures S1 and S2 allow for a deviation from the ideal octahedra of the cubic phase, with the *R*3*m* model showing two groups of three Sn–Br distances equal to each other, the *I*4*cm* model having three pairs of equal distances, and the *Pmc*2_1_ model (cf. Figure 3A) inducing a further distortion with only two distances equal to each other. The peak related to the Br–Br correlation is broader than that in the iodide counterparts, as already reported in previous characterizations, and was attributed to the low rigidity of the octahedra.^39,51^ A similar description can be applied to FASnBr_3_ (Figure S2), where the spread of distances, for the Sn–Br and Br–Br correlations, is even more pronounced, and an orthorhombic distortion is necessary to describe the crystal structure (see Figure 3B). In fact, the broadening of the first two peaks is even larger. Fits with the cubic, rhombohedral, and tetragonal models are very poor, whereas an orthorhombic distortion better reproduces the experimental trends (cf. Figure S2 and Figure 3B). All of the models selected to describe the local structure for the RT data are depicted in the top row of Figure 4.

**Figure 4:**
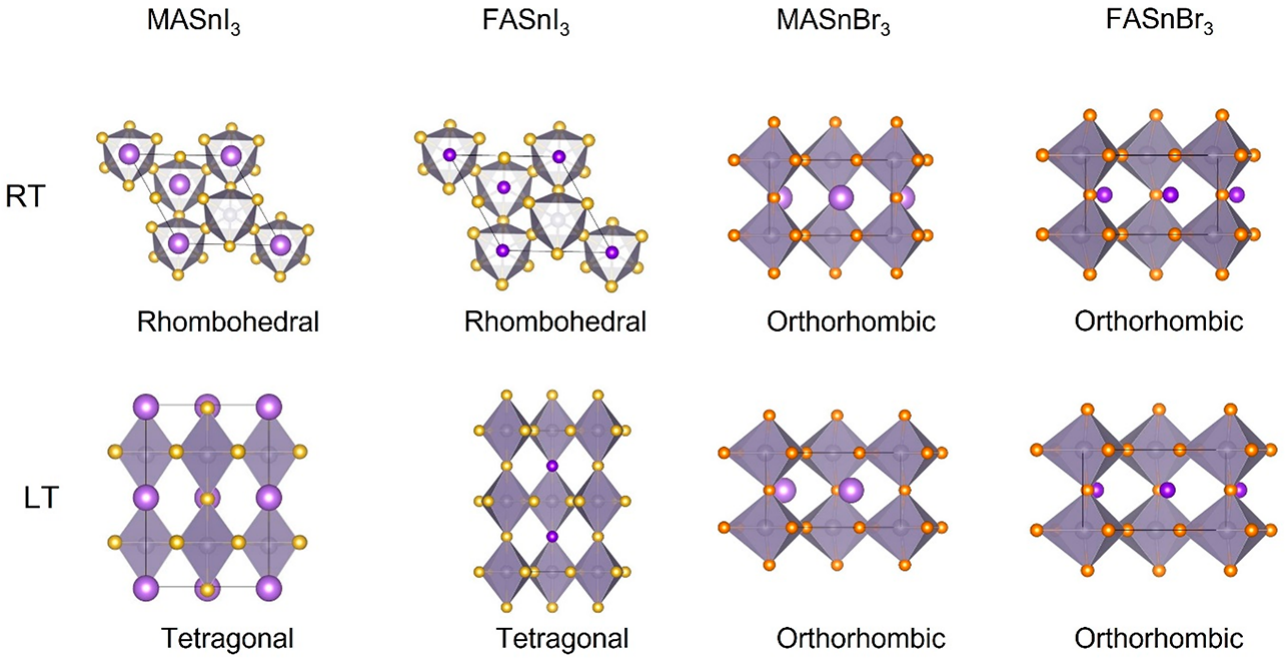
Structural sketches of the models selected to describe the local structure of ASnX_3_, where A = MA or FA and X = I or Br: MASnI_3_, MASnI_3_, MASnBr_3_, and FASnBr_3_.

When moving to the low-temperature data (80 K), we find peaks become sharper but observe a similar trend of increasing distortion by increasing the size of the A cation and the hardness of the anion (Figure 5).

**Figure 5:**
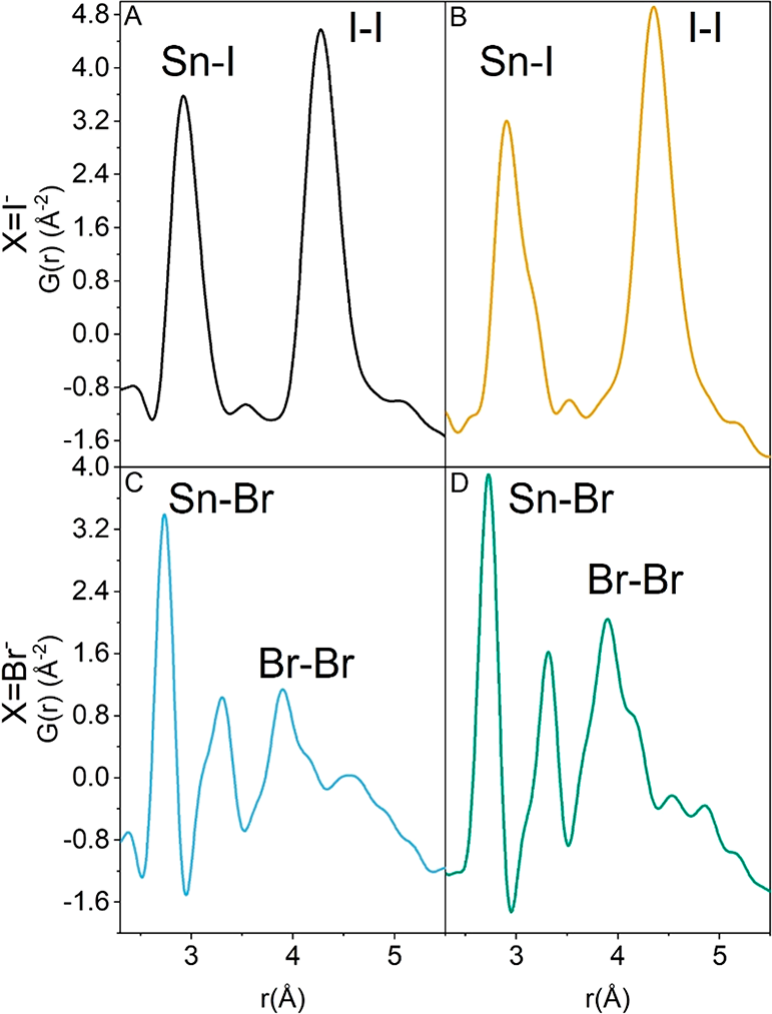
Raw X-ray PDF data collected at 80 K from ASnX_3_, where A = MA or FA and X = I or Br.

MASnI_3_ (Figure 5A) shows quite sharp peaks for the Sn–Br and Br–Br correlations, suggesting the presence of close-to-ideal octahedra. Fits with the *I*4/*mcm*, the same space group of the average structure at 80 K, *R*3*m*, and *I*4*cm* all give better results than that with *Pm*3̅*m* (Figures S4 and S5), with a slightly worse agreement with *I*4/*mcm*, suggesting that a small degree of off-centering of the B cation is present also at low temperatures. When moving to FASnI_3_ (Figure 5B), we find the first peak clearly shows asymmetry. The models tested with the room-temperature data, *Pm*3̅*m*, *R*3*m*, and *I*4*cm*, were also applied to the data at 80 K but did not yield good agreement (Figure S6). Interestingly, the *R*3*m* model, which allows for only the displacement of the B cation but not for the tilting of the octahedra, is clearly not able to reproduce the asymmetry of the Sn–Br correlations. When the tilting is included, for example with the *I*4*cm* model, the correlation is better described, suggesting that octahedral tilting might play an important role in the local structure of FASnI_3_. To further explore this hypothesis, we tested different tilts. The *P*4/*mbm* model, which corresponds to the tilt *a*^0^*a*^0^*c*^+^ in Glazer notation, does not provide a good fit to the data, whereas the *I*4/*mcm* and *Pnma* models, *a*^0^*a*^0^*c*^–^ and *a*^+^*b*^–^*b*^–^ tilts, respectively, show better agreement (Figure 6A and Figure S7).^52^ All three tilts involve a rotation on the *c*-axis parallel to the 4-fold axis; the first tilt system gives rise to layers of octahedra with exactly the same relative orientation, whereas the other two lead to layers with opposite relative orientations. This type of tilting seems to provide a better fit to the experimental trends. The likelihood of an orthorhombic distortion is also supported by the fact that the average structure at 80 K is in the *Pnma* space group^35^ and that the local structure of other MHPs, such as MAPbBr_3_ and MAPbCl_3_, was described with this symmetry.^39,40^

**Figure 6:**
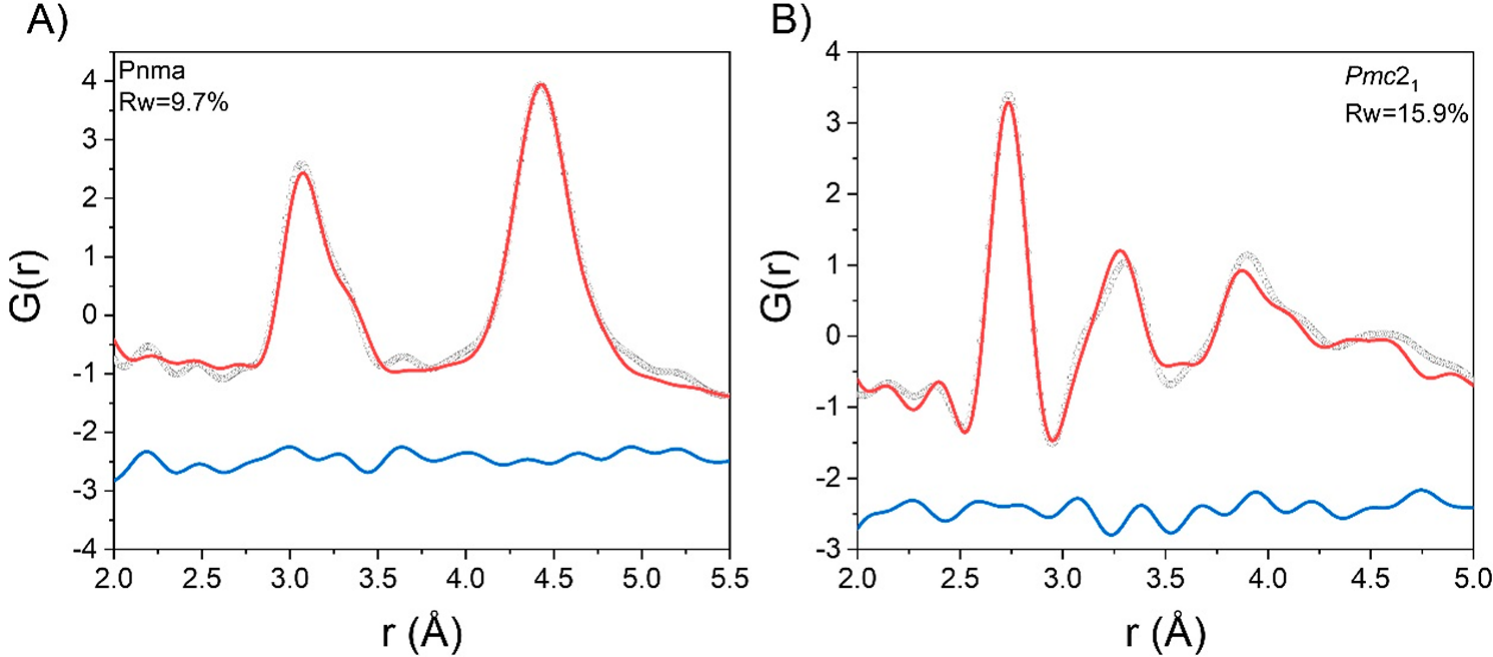
(A) Fit of the X-ray PDF data at 80 K from 2.0 to 5.5 Å against space group *Pnma* for FASnI_3_. (B) Fit of the X-ray PDF data at 80 K from 2.0 to 5.0 Å for MASnBr_3_ against the orthorhombic model. Gray dotted line, observed; red line, calculated; blue line, difference (shifted by −2.5 for ease of visualization).

The bromide compounds (Figure 5C,D), in analogy with the room-temperature data, have some distinctive features with respect to the iodide ones. The peak related to the Br–Br correlation is broader, and the signal due to the Sn–Br correlation is clearly split into two peaks, one sharper and one asymmetric, in contrast with MASnI_3_ and FASnI_3_ that show only broadening and asymmetry (Figure 5A,B). The cubic, tetragonal, and rhombohedral models cannot reproduce the experimental trend, in particular the second peak related to Sn–Br distances (Figure S8). An orthorhombic model with space group *Pmc*2_1_ shows better agreement (Figure 6B). The first sharp peak can be attributed to three Sn–Br distances close to 2.73 Å, and the second asymmetric peak to three longer distances. As expected from the trends highlighted so far, the distortion from the ideal cubic symmetry is larger in FASnBr_3_; also in this case, the cubic, tetragonal, and rhombohedral models fail to reproduce the experimental trends, whereas an orthorhombic distortion provides a better fit (Figure S9). The structures that better describe the 80 K PDF data for the four samples analyzed in this paper are summarized in the bottom row of Figure 4.

This systematic characterization of the local structure of tin halide perovskites by total scattering methods sheds light on the extension and nature of local distortions as a function of composition and temperature. Larger displacements are observed when the cation size is increased, i.e., from MA to FA, and the hardness of the anion is increased, i.e., from Br^–^ to I^–^. Furthermore, a certain degree of orthorhombicity was found to be always present in the investigated compositions at any temperature and crystal symmetry, possibly due to the hydrogen bonding with the organic group strongly affecting the local structure.^32,39−41^ This description perfectly fits the local structure of lead halide perovskites and is in good agreement with the results presented here, in particular at low temperature and for the bromide compounds, displaying greater distortion. On the contrary, at room temperature and above, the local structure of tin iodide compounds seems to be more affected by off-centering of the B-site cation due to the higher activity of the lone pair with respect to lead analogues. To assess whether the models that describe well the local structure of these MHPs also lead to a good description of the electronic structure, calculations within hybrid density functional theory (DFT) were performed (see the Supporting Information for details). The calculated band gaps are listed in Table 1 and compared with those from experiments.

**Table 1:** Band Gaps (electronvolts) Calculated for Different Models within the PBE0(α) Functional and Including the Effect of Spin–Orbit Coupling, with Experimental Values Included for Comparison.

	*Pm*3̅*m*	LT	RT	MD RT	exptl
MASnBr_3_	1.29	1.21	1.16	2.16	2.00, 2.15^53,54^
MASnI_3_	0.48	0.59	1.02	1.27	1.23^55^
FASnBr_3_	1.73	1.66	1.75	2.54	2.40^53^
FASnI_3_	0.74	0.36	1.28	1.59	1.41^56^

We first consider band gaps calculated within the *Pm3̅m* model and observe that they are significantly (by up to 0.75 eV) smaller than their experimental counterparts. This is in agreement with previous studies which have found that the perfect cubic *Pm*3̅*m* model underestimates the band gap of halide perovskites at finite temperatures.^57,58^ As shown in ref (57), the effect of thermal disorder on the band gap amounts to up to 0.8 eV in inorganic halide perovskites, which for small-band gap compounds implies an error of >50% in the final result. This is consistent with the underestimation of the band gap observed here. The comparison with experimental values is not generally better when considering the LT and RT models coming from fitting the PDF measurements. In the cases of MASnBr_3_ and FASnBr_3_, the finite-temperature LT models give band gaps even smaller than that of the perfect cubic structure. Additionally, for MASnI_3_ and FASnI_3_, we observe large differences between band gaps calculated for the LT and RT structures, not consistent with the relatively weak temperature dependence of the band gaps of halide perovskites.^59^ Finally, when comparing the values calculated for the RT models with those from experiments, we observe significant underestimation of the band gaps, especially in the cases of MASnBr_3_ and FASnBr_3._ We therefore conclude that static small united cell models, which describe the local geometry well and fit the PDF data, do not always provide a good description of the electronic structure of MHPs.

It has been shown that local dynamical distortions must be considered to accurately determine band gaps of halide perovskites at finite temperatures.^57^ Therefore, we also perform molecular dynamics (MD) simulations for the four compounds at RT (see the Supporting Information for details). We then calculate band gaps on top of 20 snapshots separated by 200 fs from MD trajectories for each compound. The resulting average values are listed in Table 1. We observe a very good agreement (within 0.2 eV) between the band gaps calculated on the basis of MD structures and their experimental counterparts, consistent with what is expected for halide perovskites.^57,58^

We now compare the structural properties extracted from MD simulations with the X-ray PDF data at room temperature. To this end, we selected 20 snapshots separated by 200 fs from each trajectory and calculated average *G*(*r*) plots for each material. The comparison between these average calculated PDFs and the experimental data for the four compounds is given in Figure 7, where only the scale factor and the unit cell parameters have been refined.

**Figure 7:**
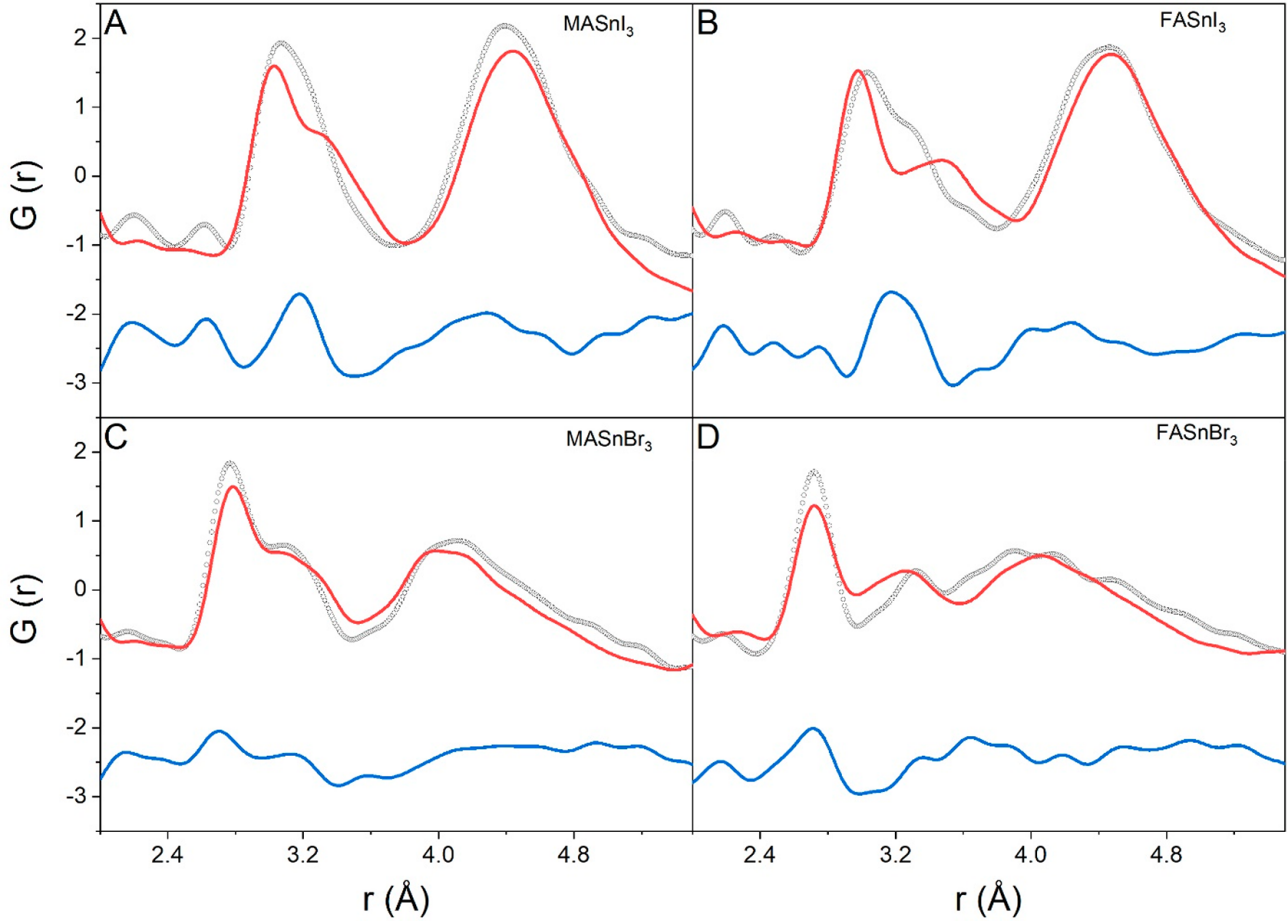
Comparison between X-ray PDF data collected at RT and MD simulations for ASnX_3_, where A = MA or FA and X = I or Br). Gray dotted line, observed; red line, calculated; blue line, difference (shifted by −2.5 for ease of visualization).

Visual inspection of the calculated and experimental data in terms of the peak number, shape, and positions indicated a fairly good agreement between the two data sets. The average PDFs from MD properly catch the main features of the experimental data, in particular the low-*r* distortion. This result supports the validity of the computational modeling and strongly indicates that a proper description of the energy gaps in tin halide perovskite requires the consideration of the local structural distortion experimentally unveiled by PDF analysis.

To summarize, we characterize by means of X-ray total scattering and PDF analysis as a function of temperature four tin halide perovskites, namely, MASnI_3_, FASnI_3_, MASnBr_3_, and FASnBr_3_. The analysis of the local structure indicates a clear trend of increasing local distortion as a function of the nature of the organic cation, halide, and temperature. In particular, larger displacements at the local scale are observed when the cation size is increased, i.e., from MA to FA, and the hardness of the anion is increased, i.e., from Br^–^ to I^–^. Electronic structure calculations have clearly shown that dynamical distortion must be considered to accurately determine the band gaps of the considered tin-based MHPs. The MD averaged local structures obtained from simulations have been compared to experimental PDFs, providing a fair agreement and thus further strengthening the correlation between electronic structure calculations and actual local structures. These results are of key importance in clarifying the structure–property correlation of lead-free MHPs, which are the most appealing materials for overcoming the toxicity issues of lead-based systems for photovoltaic devices.

## Supporting Information Available

CIF files for the final models obtained by PDF fits (ZIP)
Experimental and computational details and additional PDF fits (PDF)

## Notes

Structures used in DFT calculations and molecular dynamics trajectories can be found in 10.5281/zenodo.7607926.
