# Supplementary material for: Cubic or Not Cubic? Combined Experimental and Computational Investigation of the Short-Range Order of Tin Halide Perovskites
Source: J Phys Chem Lett. 2023 Feb 21;14(8):2178–86. doi: 10.1021/acs.jpclett.3c00105 (PMC9986956; doi:10.1021/acs.jpclett.3c00105)
Supplement: Supplementary file 2 — jz3c00105_si_002.pdf [file jz3c00105_si_002.pdf]

Supporting Information

Cubic or not Cubic? Combined Experimental and Computational Investigation of the Short-Range Order of Tin Halide Perovskites

Marta Morana,[a,] Julia Wiktor,[b] Mauro Coduri,[c] Rossella Chiara,[c] Carlotta Giacobbe,[d] Eleanor Lawrence Bright,[d] Francesco Ambrosio[e,f] Filippo De Angelis,[g,h,i] Lorenzo Malavasi[c,*]*

[a] *Department of Earth Sciences, University of Firenze, Via G. La Pira 4, 50121, Firenze, Italy*
[b]*Department of Physics, Chalmers University of Technology, 412 96 Goteborg, Sweden*
[c]*Department of Chemistry and INSTM, Viale Taramelli 16, 27100, Pavia, Italy*
[d] *ESRF, 71 Avenue des Martyrs, 38000, Grenoble, France*
[e] *Department of Chemistry and Biology "A. Zambelli", University of Salerno, Via Giovanni Paolo II 132, 84084 Fisciano, Salerno, Italy*
[f] *Dipartimento di Scienze, University of Basilicata, Viale dell'Ateno Lucano, 10, 85100 Potenza, Italy*
[g] *Computational Laboratory for Hybrid/Organic Photovoltaics (CLHYO), Istituto CNR di Scienze e Tecnologie Chimiche "Giulio Natta" (CNR-SCITEC), Via Elce di Sotto 8, 06123 Perugia, Italy*
[h] *Department of Chemistry, Biology and Biotechnology, University of Perugia, Via Elce di Sotto 8, 06123 Perugia, Italy*
[i]*Department of Natural Sciences & Mathematics, College of Sciences & Human Studies, Prince Mohammad Bin Fahd University, Dhahran 34754, Saudi Arabia*

Synthesis

The $ASnX_3$ powders (A = methylammonium, MA and formamidinium, FA; X = Br and I) were synthetized by a solution method under nitrogen flux. A stoichiometric amount of Tin(II) acetate was dissolved in a large excess of 48% w/w aqueous HBr or 57% w/w aqueous HI and 50% w/w aqueous H_3PO_2 at 101 °C under magnetic stirring. After obtaining the solid dissolution, the stoichiometric quantity of the amine was slowly added. Subsequently, the solution was cooled down to room temperature promoting the precipitation of the powdery product. The precipitate was immediately filtered, dried at 65°C under vacuum overnight and stocked in glove box

Data collection

X-ray PDF data were collected the material science ID11 beamline of the ESRF, in Grenoble, at incident wavelength at incident wavelength λ=0.158135 ˚A, using a Dectris Eiger2 X CdTe 4M detector placed 125 mm away from the sample. The distance was calibrated against a CeO2 standard using the pyFAI package, which was used also to integrate the 2D images.[1] The powdered samples were packed into quartz capillaries (provided by Hilgenberg) with a 0.5 mm diameter and rotated during the data collection. A Cryostream was employed to collect low and high temperature data from 80 to 360 K.

Data analysis

Rietveld refinement to assess the quality of the samples was performed using the software Topas.[2] PDFs were obtained using PDFgetX3 with a Q_{max} of 23.5 ˚A and PDF modelling was carried out using PDFGui.[3,4] The instrumental parameters used in the PDF fit were Q_{broad}= 0.03 Å and Q_{damp}=0.025 Å, as determined from the CeO_2 standard. The organic cations in the A site were modeled as a pseudoatom with an equivalent scattering power, using K for CH_3NH_3 and Mn for $CH(NH_2)_2$). Starting coordinates in the different models were obtained from the cubic structure using the ”TRANSTRU” tool on the Bilbao Crystallographic Server.[5–7]

Density functional theory and molecular dynamics simulations

Density functional theory (DFT) calculations are performed within the CP2K code using the SCAN functional for energy and force calculations.[8–10] We use a double-ζ basis set (DZVP-MOLOPT) and the norm-conserving Goedecker–Teter–Hutter (GTH) pseudopotentials.[11,12] A cutoff of 600 Ry is set for the expansion of the electron density. The calculations are carried out using supercells with the Brillouin zone sampled only at the Γ point. For each compound, we consider three models, the *Pm*-3*m* cubic, the low temperature (LT), and room temperature (RT) structures, corresponding to the phases shown in Fig. 4 of the main text. The supercells contain from 768 to 864 atoms. We fix the lattice parameters and atomic positions of the Sn, Br, and I atoms to the ones obtained from the fits to the X-ray PDF data and fully relax the FA and MA molecules in the models.

In band gap calculations, we use Koopmans-compliant PBE0(α) functionals, that have been shown to reproduce experimental results well, as long as all relevant effects, like spin-orbit coupling or disorder, are taken into account.[13,14] To reduce the computational cost of hybrid DFT calculations in large supercells, we employ the auxiliary density matrix method (ADMM) as implemented in the CP2K code.[15] To determine the mixing parameter α in the hybrid functional, for each compound in the *Pm*-3*m* model, we remove a single halide atom from the supercell, thus creating a halide vacancy V_X, and introducing a localized state in the band gap of the perovskite, as done in Ref. 14. Then, we calculate the single-particle energy level for both the occupied (neutral supercell, V_X^0) and unoccupied (positively charged, V_X^+) states at the PBE0(α) level, considering three different values of α (0.25, 0.30, 0.35). The unoccupied levels are corrected according to the method by Falletta *et al.*[16] The intersection between the linear evolution of the energy levels for occupied and empty states corresponds to the value of α fulfilling the Koopmans condition, i.e. the energy level of a single-particle state is independent from electron occupation.[17–19]

To make comparison with experiments, we also include the effect of spin-orbit coupling on the band gaps calculated in all models. To determine $\Delta_{\mathrm{SOC}}= E_{\mathrm{gap}}^{\mathrm{SOC}} - E_{\mathrm{gap}}^{\mathrm{no\ SOC}}$, we compare band gap values calculated for the *Pm*-3*m* model of each compound with and without spin-orbit interactions. These calculation are performed in the VASP code.[20–22] In Table S1 we give the values for Δ_{SOC}, together with uncorrected values of $E_{\mathrm{gap}}^{\mathrm{no\ SOC}}$ calculated at the PBE0(α) levels of theory.

Molecular dynamics (MD) simulations of the four compounds at 293 K were performed with temperature controlled by the Nose-Hoover thermostat.[23,24] The runs are carried out at constant

pressure using an isotropic cell.[25] The timestep is set to 1 fs and for each material we carry out the MD simulation for 5 ps. The first 1 ps is considered as equilibration and is excluded from the statistics. The simulations are carried out with the CP2K code, The computational setup is the same employed for structural calculations at the SCAN level. From each trajectory, we select 20 snapshots separated by 200 fs , to calculate band gaps at the PBE0(α) level (MD-RT in Table S1).

The resulting structural models were employed to compare the outcome of the simulations with the experimental PDFs using the PDFGui.[4] The phase scale factor for each model was fixed at 0.05 and the unit cell parameters were set to be same and then refined against the experimental data together with the data scale factor.

	$E_{\mathrm{gap}}^{\mathrm{no\ SOC}}$ (eV)				α (%)	Δ_{SOC} (eV)
	Pm*3̄*m	**LT**	**RT**	**MD RT**		
**$MASnBr_3$**	1.63	1.55	1.50	2.50	20	−0.34
**$MASnI_3$**	0.84	0.95	1.38	1.63	14	−0.36
**$FASnBr_3$**	1.98	1.91	2.00	2.79	24	−0.25
**$FASnI_3$**	1.06	0.68	1.60	1.91	19	−0.32

Table S1. Band gaps calculated for different models within the PBE0(α) functional, the fraction of the exact exchange used in the PBE0(α) method and the size of the band gap reduction due to SOC, Δ_{SOC}.

Additional Figures

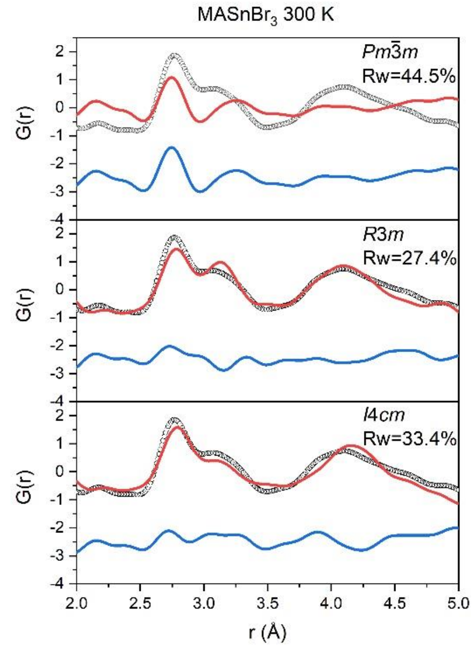

Figure S1. Fits of the X-ray PDF data at 300 K from 2.0 to 5.0 Å against the space groups *Pm*-3*m*, *R*3*m*, and *I*4*cm* for $MASnBr_3$. Gray dotted line: observed; red line: calculated; blue line: difference.

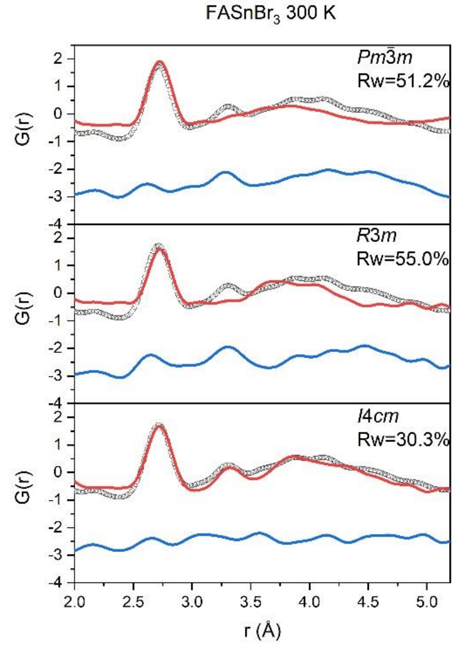

Figure S2. Fits of the X-ray PDF data at 300 K from 2.0 to 5.0 Å against the space groups *Pm*-3*m*, *R*3*m*, and *I*4*cm* for $FASnBr_3$. Gray dotted line: observed; red line: calculated; blue line: difference.

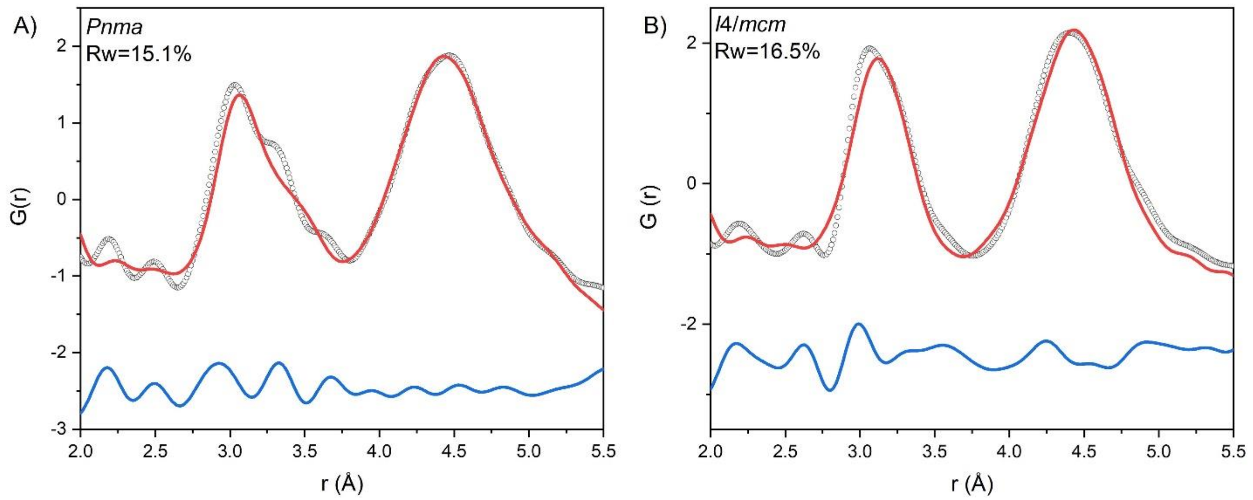

Figure S3. Fits of the X-ray PDF data at 300 K from 2.0 to 5.5 Å against the space group *Pnma* for $FASnI_3$ (a) and *I*4/*mcm* for $MASnI_3$ (b). Gray dotted line: observed; red line: calculated; blue line: difference.

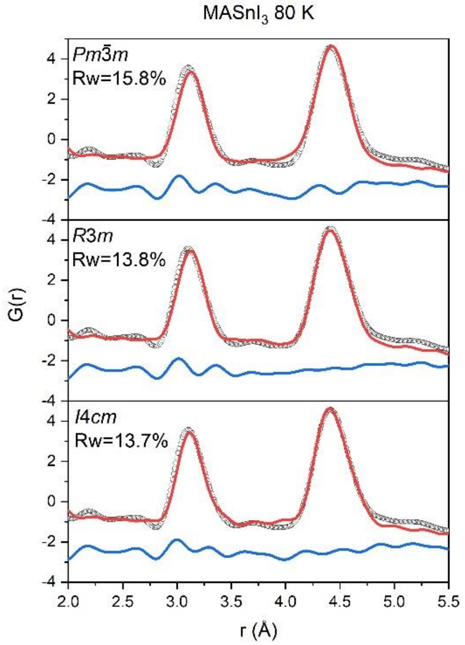

Figure S4. Fits of the X-ray PDF data at 80 K from 2.0 to 5.5 Å against the space groups *Pm*-3*m*, *R*3*m*, and *I*4*cm*. for $MASnI_3$. Gray dotted line: observed; red line: calculated; blue line: difference.

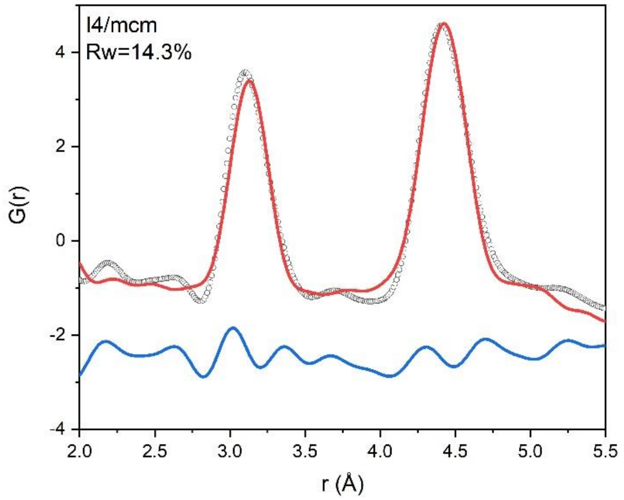

Figure S5. Fits of the X-ray PDF data at 80 K from 2.0 to 5.5 Å against the space group *I*4/*mcm* for $MASnI_3$. Gray dotted line: observed; red line: calculated; blue line: difference.

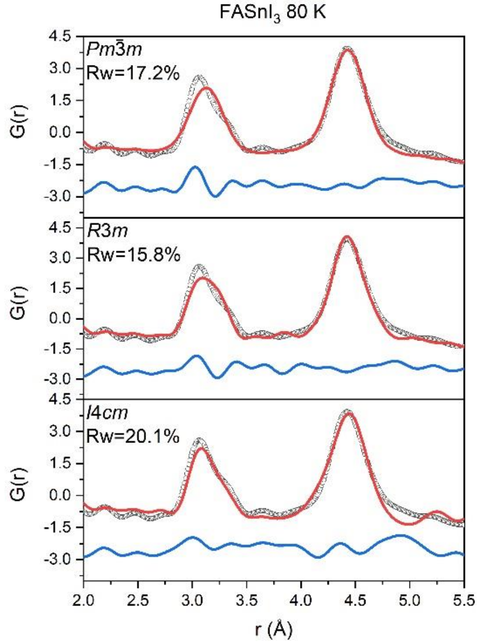

Figure S6. Fits of the X-ray PDF data at 80 K from 2.0 to 5.5 Å against the space groups *Pm*-3*m*, *R*3*m*, and *I*4*cm*. for $FASnI_3$. Gray dotted line: observed; red line: calculated; blue line: difference.

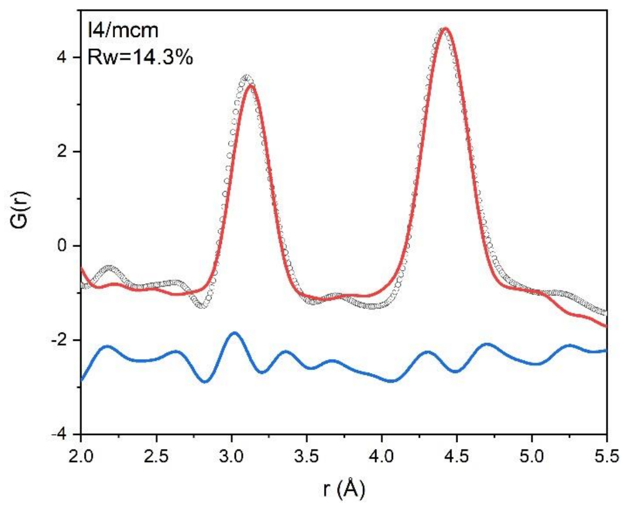

Figure S7 Fit of the X-ray PDF data at 80 K from 2.0 to 5.5 Å against the space group *I*4/m*cm*. for $FASnI_3$. Gray dotted line: observed; red line: calculated; blue line: difference.

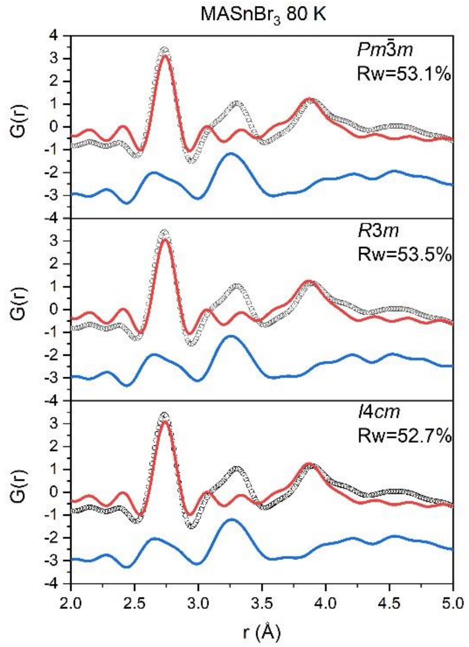

Figure S8. Fits of the X-ray PDF data at 80 K from 2.0 to 5.0 Å against the space groups *Pm*-3*m*, *R*3*m*, and *I*4*cm*. for $MASnBr_3$. Gray dotted line: observed; red line: calculated; blue line: difference.

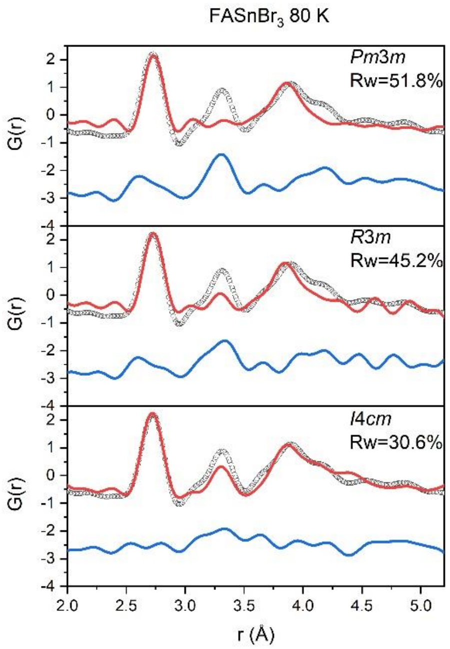

Figure S9. Fits of the X-ray PDF data at 80 K from 2.0 to 5.0 Å against the space groups *Pm*-3*m*, *R*3*m*, and *I*4*cm*. for $FASnBr_3$. Gray dotted line: observed; red line: calculated; blue line: difference.

References

(1) Kieffer, J.; Valls, V.; Blanc, N.; Hennig, C. New Tools for Calibrating Diffraction Setups. *J Synchrotron Rad* **2020**, *27* (2), 558–566. https://doi.org/10.1107/S1600577520000776.

(2) Coelho, A. A. *TOPAS* and *TOPAS-Academic* : An Optimization Program Integrating Computer Algebra and Crystallographic Objects Written in C++. *J Appl Crystallogr* **2018**, *51* (1), 210–218. https://doi.org/10.1107/S1600576718000183.

(3) Juhás, P.; Davis, T.; Farrow, C. L.; Billinge, S. J. L. *PDFgetX3* : A Rapid and Highly Automatable Program for Processing Powder Diffraction Data into Total Scattering Pair Distribution Functions. *J Appl Crystallogr* **2013**, *46* (2), 560–566. https://doi.org/10.1107/S0021889813005190.

(4) Farrow, C. L.; Juhas, P.; Liu, J. W.; Bryndin, D.; Božin, E. S.; Bloch, J.; Proffen, T.; Billinge, S. J. L. PDFfit2 and PDFgui: Computer Programs for Studying Nanostructure in Crystals. *J. Phys.: Condens. Matter* **2007**, *19* (33), 335219. https://doi.org/10.1088/0953-8984/19/33/335219.

(5) Aroyo, M. I.; Perez-Mato, J. M.; Orobengoa, D.; Tasci, E. Crystallography Online: Bilbao Crystallographic Server.

(6) Aroyo, M. I.; Kirov, A.; Capillas, C.; Perez-Mato, J. M.; Wondratschek, H. Bilbao Crystallographic Server. II. Representations of Crystallographic Point Groups and Space Groups. *Acta Crystallogr A Found Crystallogr* **2006**, *62* (2), 115–128. https://doi.org/10.1107/S0108767305040286.

(7) Aroyo, M. I.; Perez-Mato, J. M.; Capillas, C.; Kroumova, E.; Ivantchev, S.; Madariaga, G.; Kirov, A.; Wondratschek, H. Bilbao Crystallographic Server: I. Databases and Crystallographic Computing Programs. *Zeitschrift für Kristallographie - Crystalline Materials* **2006**, *221* (1), 15–27. https://doi.org/10.1524/zkri.2006.221.1.15.

(8) Kühne, T. D.; Iannuzzi, M.; Del Ben, M.; Rybkin, V. V.; Seewald, P.; Stein, F.; Laino, T.; Khaliullin, R. Z.; Schütt, O.; Schiffmann, F.; Golze, D.; Wilhelm, J.; Chulkov, S.; Bani-Hashemian, M. H.; Weber, V.; Borštnik, U.; Taillefumier, M.; Jakobovits, A. S.; Lazzaro, A.; Pabst, H.; Müller, T.; Schade, R.; Guidon, M.; Andermatt, S.; Holmberg, N.; Schenter, G. K.; Hehn, A.; Bussy, A.; Belleflamme, F.; Tabacchi, G.; Glöß, A.; Lass, M.; Bethune, I.; Mundy, C. J.; Plessl, C.; Watkins, M.; VandeVondele, J.; Krack, M.; Hutter, J. CP2K: An Electronic Structure and Molecular Dynamics Software Package - Quickstep: Efficient and Accurate Electronic Structure Calculations. *J. Chem. Phys.* **2020**, *152* (19), 194103. https://doi.org/10.1063/5.0007045.

(9) Hutter, J.; Iannuzzi, M.; Schiffmann, F.; VandeVondele, J. CP2K: Atomistic Simulations of Condensed Matter Systems: CP 2 K Simulation Software. *WIREs Comput Mol Sci* **2014**, *4* (1), 15–25. https://doi.org/10.1002/wcms.1159.

(10) Sun, J.; Ruzsinszky, A.; Perdew, J. P. Strongly Constrained and Appropriately Normed Semilocal Density Functional. *Phys. Rev. Lett.* **2015**, *115* (3), 036402. https://doi.org/10.1103/PhysRevLett.115.036402.

(11) VandeVondele, J.; Hutter, J. Gaussian Basis Sets for Accurate Calculations on Molecular Systems in Gas and Condensed Phases. *The Journal of Chemical Physics* **2007**, *127* (11), 114105. https://doi.org/10.1063/1.2770708.

(12) Goedecker, S.; Teter, M.; Hutter, J. Separable Dual-Space Gaussian Pseudopotentials. *Phys. Rev. B* **1996**, *54* (3), 1703–1710. https://doi.org/10.1103/PhysRevB.54.1703.

(13) Miceli, G.; Chen, W.; Reshetnyak, I.; Pasquarello, A. Nonempirical Hybrid Functionals for Band Gaps and Polaronic Distortions in Solids. *Phys. Rev. B* **2018**, *97* (12), 121112. https://doi.org/10.1103/PhysRevB.97.121112.
(14) Bischoff, T.; Wiktor, J.; Chen, W.; Pasquarello, A. Nonempirical Hybrid Functionals for Band Gaps of Inorganic Metal-Halide Perovskites. *Phys. Rev. Materials* **2019**, *3* (12), 123802. https://doi.org/10.1103/PhysRevMaterials.3.123802.
(15) Guidon, M.; Hutter, J.; VandeVondele, J. Auxiliary Density Matrix Methods for Hartree−Fock Exchange Calculations. *J. Chem. Theory Comput.* **2010**, *6* (8), 2348–2364. https://doi.org/10.1021/ct1002225.
(16) Falletta, S.; Wiktor, J.; Pasquarello, A. Finite-Size Corrections of Defect Energy Levels Involving Ionic Polarization. *Phys. Rev. B* **2020**, *102* (4), 041115. https://doi.org/10.1103/PhysRevB.102.041115.
(17) Janak, J. F. Proof That ∂ E ∂ n i = ε in Density-Functional Theory. *Phys. Rev. B* **1978**, *18* (12), 7165–7168. https://doi.org/10.1103/PhysRevB.18.7165.
(18) Perdew, J. P.; Parr, R. G.; Levy, M.; Balduz, J. L. Density-Functional Theory for Fractional Particle Number: Derivative Discontinuities of the Energy. *Phys. Rev. Lett.* **1982**, *49* (23), 1691–1694. https://doi.org/10.1103/PhysRevLett.49.1691.
(19) Yang, W.; Zhang, Y.; Ayers, P. W. Degenerate Ground States and a Fractional Number of Electrons in Density and Reduced Density Matrix Functional Theory. *Phys. Rev. Lett.* **2000**, *84* (22), 5172–5175. https://doi.org/10.1103/PhysRevLett.84.5172.
(20) Blöchl, P. E. Projector Augmented-Wave Method. *Phys. Rev. B* **1994**, *50* (24), 17953–17979. https://doi.org/10.1103/PhysRevB.50.17953.
(21) Kresse, G.; Hafner, J. *Ab Initio* Molecular Dynamics for Liquid Metals. *Phys. Rev. B* **1993**, *47* (1), 558–561. https://doi.org/10.1103/PhysRevB.47.558.
(22) Kresse, G.; Furthmüller, J. Efficient Iterative Schemes for *Ab Initio* Total-Energy Calculations Using a Plane-Wave Basis Set. *Phys. Rev. B* **1996**, *54* (16), 11169–11186. https://doi.org/10.1103/PhysRevB.54.11169.
(23) Nosé, S. A Unified Formulation of the Constant Temperature Molecular Dynamics Methods. *The Journal of Chemical Physics* **1984**, *81* (1), 511–519. https://doi.org/10.1063/1.447334.
(24) Hoover, W. G. Canonical Dynamics: Equilibrium Phase-Space Distributions. *Phys. Rev. A* **1985**, *31* (3), 1695–1697. https://doi.org/10.1103/PhysRevA.31.1695.
(25) Martyna, G. J.; Tobias, D. J.; Klein, M. L. Constant Pressure Molecular Dynamics Algorithms. *The Journal of Chemical Physics* **1994**, *101* (5), 4177–4189. https://doi.org/10.1063/1.467468.
